# Levothyroxine treatment and gastric juice pH in humans: the proof of concept

**DOI:** 10.1007/s12020-022-03056-1

**Published:** 2022-04-27

**Authors:** Camilla Virili, Giovanni Bruno, Maria Giulia Santaguida, Lucilla Gargano, Ilaria Stramazzo, Corrado De Vito, Alessia Cicenia, Giulia Scalese, Barbara Porowska, Carola Severi, Marco Centanni

**Affiliations:** 1 Department of Medico-surgical Sciences and Biotechnologies, “Sapienza” University of Rome, Latina, Italy; 2 Department of Translational and Precision Medicine, Gastroenterology Unit, ‘Sapienza” University of Rome, Rome, Italy; 3 Endocrine Unit, AUSL Latina, Latina, Italy; 4 Department of Public Health and Infectious Diseases, University of Rome, Rome, Italy; 5 General and Specialistic Surgery ‘Paride Stefanini’, University of Rome, Rome, Italy

**Keywords:** Hypothyroidism, Levothyroxine dose, Levothyroxine malabsorption, Gastric juice pH, *Helicobacter pylori*, Atrophic gastritis

## Abstract

**Purpose:**

Despite the absorption of oral thyroxine (T4) occurs in the small bowel, several patients with gastric disorders show an increased need for T4. In vitro evidence suggested that medium pH variations interfere with T4 dissolution. This study was aimed at finding the proof of concept of a direct relationship between the minimal effective dose of T4 and the actual gastric juice pH.

**Patients and methods:**

Among 311 consecutively thyroxine-treated patients, 61 bearing Hashimoto’s thyroiditis (52 F/9 M; median age = 51 years) who complained persistent dyspepsia and/or upper abdominal symptoms following a noninvasive workup for gastrointestinal disorders, underwent EGDS with multiple biopsies and gastric juice pH measurement. All patients accepted to take thyroxine in fasting conditions, abstaining from eating or drinking for one hour.

**Results:**

Thyroxine requirement increased along with the rising gastric pH (*ρ* = 0.4229; *p* = 0.0007). A multivariate analysis revealed that gastric pH was, beside body mass index, the far more important independent variable in determining the effective dose of T4 (*p* = 0.001). The ROC curve revealed that the pH threshold for an increased thyroxine requirement was at 2.28, being the AUC by 78%. Subdividing patients by the histologic findings, it appeared a significant increase (*p* = 0.0025) along with the progressive damage of gastric mucosa.

**Conclusion:**

The in vivo measurement of gastric pH highlighted its key role in determining the minimal effective dose of oral T4 and may explain the interference of food, of some drugs and gut disorders on levothyroxine treatment

## Introduction

In the era of Precision Medicine [1] the factors affecting the minimal effective dose of any drug must be considered when treating patients. Levothyroxine sodium tablet ranks among the most prescribed drugs worldwide and represents a critical-dose drug, as little variations in the blood concentration may cause treatment failure, as well as iatrogenic thyrotoxicosis [2–5]. Despite its narrow therapeutic window imposes a careful dose titration, an estimated rate of 30–60% treated patients still shows an out-of-target TSH [6], and side effects are described [7, 8]. An individualized thyroxine treatment is therefore advisable and requires the knowledge of thyroxine biochemical and pharmacologic characteristics [9–13] as well as patients’ anthropometric data and habits [14–16]. The lean body mass is a major determinant of thyroxine dose [14] but, in clinical practice, the physicians usually choose the dose based on patient weight or BMI [5]. Furthermore, to obtain the therapeutic effect with the minimal effective dose of T4, physicians should also take into account the use of interfering foods and drugs [17, 18].

Oral thyroxine preparation is a sodium salt whose linear absorption rate in the blood lasts 60 to 90 min [19], then reaching a plateau. A seminal paper from Hays MT, demonstrated that the absorption of oral thyroxine occurs in the small bowel with different percentages [11]. Therefore, that thyroxine malabsorption may occur in patients with celiac disease, lactose intolerance, short bowel syndrome, or giardiasis is somewhat expected [17]. Nevertheless, a key role for the stomach in the oral T4 absorption process has also been suggested on a clinical ground, since several patients with gastric disorders show an increased need for thyroxine. Evidence of an increased T4 requirement has been provided in patients with *Helicobacter pylori* [*H. pylori*] infection [20, 21], chronic atrophic gastritis [21], and gastroparesis [22] as well as in patients chronically treated with proton pump inhibitors [23]. Most of these conditions feature an altered gastric juice pH since the different gastritis patterns can deeply affect gastric acid output mainly when active inflammation [i.e. *H. pylori* infection] or atrophy involve the acid-producing oxyntic glands [24–28]. As described for other drugs [29], gastric pH has been suggested to be a critical factor for both disaggregation and dissolution of thyroxine [30, 31]. However, the proof of concept of a relationship between the minimal effective dose of T4 and the actual gastric pH needs a direct assessment and this represented the aim of our study. Primary outcome of the study was to correlate pH values of fasting gastric acid secretion with the dose required to obtain target serum TSH in thyroxine-treated hypothyroid patients.

## Patients and methods

### Patients

**Fig. 1:**
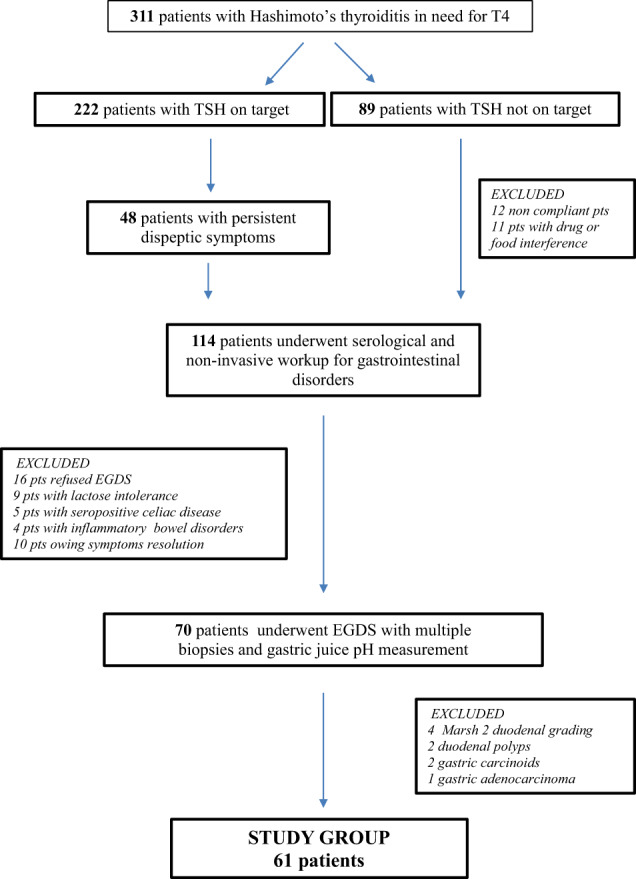
Flow chart of patients’ selection

Patients were recruited in a tertiary Endocrinology unit between 2018 and 2020 among a sequentially examined cohort of hypothyroid outpatient, with a definite diagnosis of Hashimoto’s thyroiditis, in need for levothyroxine treatment according to the ATA guidelines (*n* = 311) [3] (Fig. 1). Patients with goiter and/or who underwent thyroid surgery and/or in follow-up for thyroid cancer, with non-thyroidal illnesses or severe chronic diseases, obese (BMI > 30 kg/m^2^), with already known diagnosis of intestinal disorders and/or previous GI surgery and pregnant or nursing were not enrolled. Furthermore, patients not compliant with levothyroxine treatment schedule, with supervening diagnosis of intestinal and/or neoplastic disorders and/or in need for not replaceable substances or drugs, known to interfere with thyroxine metabolism/absorption (see for rev ref [18]) have been progressively excluded from the study (Fig. 1).

#### Design of the study

Overall, 222 out of 311 patients reached the target TSH (0.8–2.5 mU/l) within the second control visit with a median thyroxine dose of 1.31 µg/kg body weight. Among these patients reaching target serum TSH, 48 complained persistent dyspepsia and/or upper abdominal symptoms, thus needing a gastroenterological evaluation (Fig. 1). Among the 89 patients not reaching target TSH, the dose of T4 has been progressively increased, until attaining the desired TSH value. However, 23 patients were excluded because of non-mendable interference issues or ascertained noncompliance (Fig. 1), while the other 66 patients underwent gastroenterological evaluation due to the persistent dyspepsia and/or the upper abdominal symptoms. Therefore, one hundred and fourteen patients underwent diagnostic workup for gastrointestinal diseases in a Gastroenterological referral center. At the end of noninvasive diagnostic workup, 44 patients were excluded from the study (18 patients with malabsorptive or inflammatory intestinal disorders, 16 refusing endoscopy, and ten patients owing symptoms resolution) (Fig. 1). The remaining 70 patients, needed to complete the diagnostic workup because of long-lasting dyspepsia or upper abdominal symptoms, persisting despite an appropriate treatment [32]. These symptoms might have occurred isolated or concomitant with chronic unexplained micro– and macrocytic anemia and/or long lasting or refractory gastrointestinal reflux disease [32, 33]. These patients underwent upper endoscopy with multiple biopsies and gastric juice pH evaluation. The protocol was consistent with the principles of the Declaration of Helsinki and the study has been performed within the usual diagnostic workup of each single patient, upon written informed consent. The study was approved by the Sapienza University Ethical Committee. Upon endoscopic evaluation, nine additional patients were excluded (see Fig. 1). Therefore, our final study group consisted in 61 patients (52 F/9 M; median age = 51 years; IQR = 41–65 years). STROBE guidelines were followed to describe this study.

#### Standardization of thyroxine treatment

Following the initial clinical examination, patients have been treated with the same brand of levothyroxine sodium in tablet formulation with an age- and weight-tailored dose. As previously described [34, 35], all subjects were requested to follow a tight schedule of thyroxine ingestion, ensuring to take the tablet while in fasting condition and then abstaining from eating or drinking anything other than water for at least 1 h after T4 ingestion [16, 19]. Patient’s compliance with treatment schedule was checked by a questionnaire, accepted at the first visit and confirmed at every examination. Following a starting T4 dose of 1.0 µg/kg body weight/day, dose adjustments were made based on TSH levels in each patient and tested every three months (median follow-up = 34 months). Once reached target TSH, stability of its value had been confirmed at least in two TSH measurements.

A reference group was used to identify a threshold beyond whom an increased need for thyroxine in hypothyroid patients could be defined. This reference group consists in 140 hypothyroid with similar anthropometric characteristics, who followed exactly the same schedule of treatment with the same brand of T4. These patients met all the criteria described for the study group and chiefly they did not show evidence of signs or symptoms of malabsorption nor dyspepsia, as previously described [34, 35]. The median daily dose of T4 required to obtain the target TSH (0.8–2.5 mU/l) in this reference group was 1.27 µg/kg body weight (IQR = 1.19–1.33) or a mean dose of 1.27 ± 0.12 (SD) µg/kg body weight. Therefore, the normal thyroxine requirement was calculated considering the mean daily dose of T4 in our reference group to which 1.96 standard deviations were added. Hence, in our hypothyroid patients, we considered increased a need for thyroxine overcoming 1.51 µg/kg body weight/day.

## Methods

### Thyroid diagnostic workup

The diagnosis of Hashimoto’s thyroiditis was based on the presence of high titer anti-thyroperoxidase antibodies [anti-TPO Abs], a characteristic ultrasonographic pattern and hypothyroidism [serum TSH > 10 mU/l]. Serum TSH and FT4 and anti-TPO Abs levels were assayed by commercial assays [Thermo Scientific, BRAHMS TSH RIA, Germany].

### Gastrointestinal diagnostic workup

As a policy of our Center, all patients undergo an accurate anamnestic interview and those with clinical suspicion and gastrointestinal signs or symptoms are referred to the abovementioned Gastroenterological center to enter a diagnostic workup, as previously described [17]. As shown in Fig. 1, 114 patients underwent a noninvasive gastroenterological screening. Briefly, the presence of lactose intolerance was evaluated by lactose H2 breath test; the first line of celiac disease investigation was based on the Elisa measurement of serum anti-transglutaminase autoantibodies (AXA Diagnostics Srl, Pomezia, Italy; cut-off title <10 U/ml). Serum gastrin 17 levels were assayed by chemiluminescence assay (Biohit Healthcare, Helsinki, Finland normal range, 13–115 pg/ml) and serum anti-parietal cells antibodies were measured by Autoantibody test system (A. Menarini Diagnostics s.r.l.—Firenze, Italy; cut-off title< 40).

### Upper endoscopy

All patients underwent upper endoscopy in the morning, fasting from at least eight hours. All endoscopy procedures were performed between 8:00 and 10:00 a.m. All subjects were sedated using intravenous midazolam [range to 2–5 mg]. Following exclusion of macroscopic gastro-duodenal lesions, 5 biopsies were collected in the stomach using a standard biopsy forceps, two from antrum, one from the *incisura angularis*, two from mid-body and two from fundus during retroversion, in accordance with the updated Sydney System [36].

### Gastric juice aspiration and gastric pH value assessment

Immediately after the insertion of the endoscope into the stomach, 5 mL of gastric juice were aspirated in fundus and mid-body [greater curve] by means of a sterile teflon catheter and collected into a sterile trap connected with the suction line of the endoscope. After collection, the pH of gastric juice was measured using a glass electrode pH–meter and afterwards titrated with a 1 N solution of NaOH to evaluate the actual H+ concentration in each sample.

### Histologic assessment

All gastric and duodenal biopsies were fixed and stained with haematoxylin and eosin for histopathologic examination. Modified Giemsa staining was used to evaluate *H. pylori* infection. Assessment of gastritis patterns was done according to the updated Sydney scoring system [36] [0 = absence, 1 = mild, 2 = moderate, 3 = severe] evaluating the acute inflammation (neutrophil infiltrate), chronic inflammation (mononuclear cell infiltrate), glandular atrophy, intestinal metaplasia, Helicobacter pylori infection [36]. Atrophy of the fundic mucosa was defined as focal or complete replacement of oxyntic glands by metaplastic pyloric or intestinal glands. An expert pathologist blinded for patient’s clinical diagnosis performed the histopathologic examination. Based on histological reports, patients were divided in 5 groups. Group 0 when no alterations in the whole gastric mucosa were found; Group 1, when only antral superficial gastritis was present; Group 2, when a superficial chronic pangastritis was described; Group 3, in the presence of active superficial pangastritis; Group 4, when atrophic chronic gastritis was found.

## Statistical analysis

A descriptive analysis was performed calculating median and interquartile range (IQR) for continue variables and absolute and relative frequencies for qualitative variables. Chi-squared and Fisher’s tests were used to compare proportions of different groups. After testing for normality with Shapiro-Wilk test, Student’s *t* test and Pearson r test or analogous nonparametric Mann–Whitney and Spearman *ρ* tests were used to compare different groups or assess correlation between variables, respectively. To assess the role of pH in determining thyroxine dose, a multiple regression analysis was built, including, beside pH, also age, sex, BMI, TSH, FT4, histologic pattern of gastritis and histologic detection of *H. Pylori*. For purpose of analysis, given the skewed distribution of the thyroxine dose, generalized linear regression analysis was carried out. Coefficient, standard error (SE) and *p* values were calculated for each of the independent variables. Statistical significance was defined as a two-sided *p* value < 0.05 for all analyses, which were carried out using STATA statistical software, version 15 (Stata Corp., College Station, TX, USA). Prof. Corrado De Vito, coauthor of the manuscript, analyzed the data.

## Results

The anthropometric and thyroid biochemical features in the whole study group has been summarized in Tables 1a and 1b. Median pH in gastric juice in the whole sample was 2.28 (IQR = 1.47–6.4) and median H^+^ concentration was 52 mEq/l (IQR = 5–76). In the whole sample the median dose of T4 required to obtain the desired TSH was 100 μg/day (IQR = 86–125) that following normalization by individual weights was equal to 1.47 μg/kg body weight/day (IQR = 1.32–1.72).

**Table 1:** **a** Anthropometric and biochemical features in the whole sample. **b** Characteristics of patients subdivided by gastric juice pH value

(a)							
Patients (*n*)	Age (years)	Sex (M/F)	Weight (kg)	Height (m)	BMI (kg/m^2^)	TSH (mU/l)	FT_4_ (ng/dl)
61	51 (41–65)	9/52	64 (59–74)	1.62 (1.57–1.66)	25.8 (22.4–29.3)	1.30 (0.85–1.94)	1.24 (1.14–1.32)

(b)			
	Patients with gastric juice pH ≤2 GROUP A	Patients with gastric juice pH > 2 GROUP B	*p*
Patients (*n*)	29	32	
Age (years)	50 (43–62)	51 (41–67)	0.7671*
Sex (M/F)	7/22	2/30	0.0712**
Weight (kg)	66 (60–77)	64 (59–74)	0.9194*
BMI (kg/m^2^)	25.8 (22.5–28.3)	26.0 (22.3–29.8)	0.6062*
TSH (mU/l)	1.56 (0.82–2-21)	1.29 (0.97–1.51)	0.1999*
FT_4_	1.21 (1.10–1.28)	1.29 (1.17–1.40)	0.0803*

Data are expressed as median values (IQs)

*Mann–Whitney test

**Fisher’s exact test

**Fig. 2:**
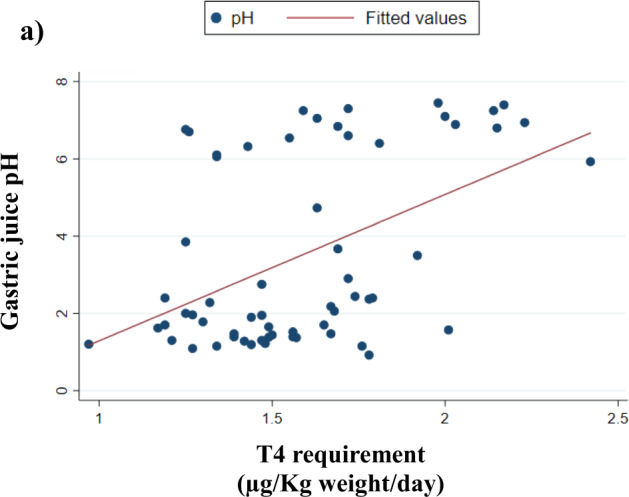
Correlation between gastric juice pH and the minimal effective dose of thyroxine

A direct correlation between the required T4 dose and an increased pH has been observed in the whole sample (*ρ* = 0.4229; *p* = 0.0007) (Fig. 2a). This finding was confirmed by the inverse correlation between the dose of T4 and the actual H^+^ juice concentration (*ρ* = −0.3987; *p* = 0.0018).

When analyzing the whole sample of the patients, the expected minimal effective dose (1.27 ± 0.24 μg/kg/day), as in the reference group, was sufficient to obtain the target serum TSH in 36 patients. The remaining 25 patients required a higher T4 dose ranging from 1.55 to 2.42 μg/kg/day. We further analyzed the dose of thyroxine by subdividing our whole sample based on gastric pH in two groups: group A (*n* = 29) with pH ≤2.0, representing the normal fasting pH in healthy human stomach [37–39], and group B (*n* = 32) with pH >2.0. As shown in Table 1b, the anthropometric characteristics and thyroid function tests were similar in these two groups.

**Fig. 3:**
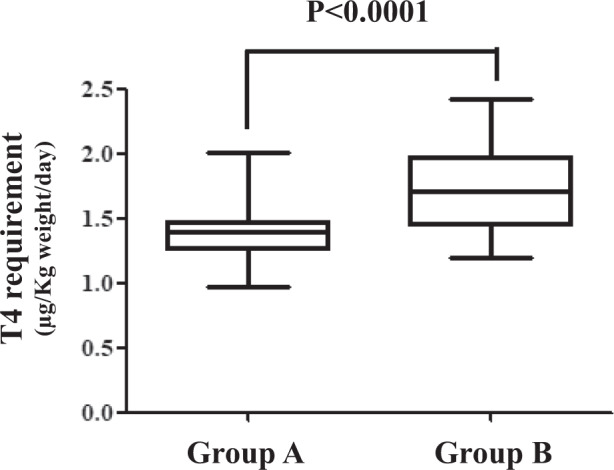
Daily thyroxine requirement in patients with gastric juice pH below/equal (Group A) or over (Group B) 2.0

**Fig. 4:**
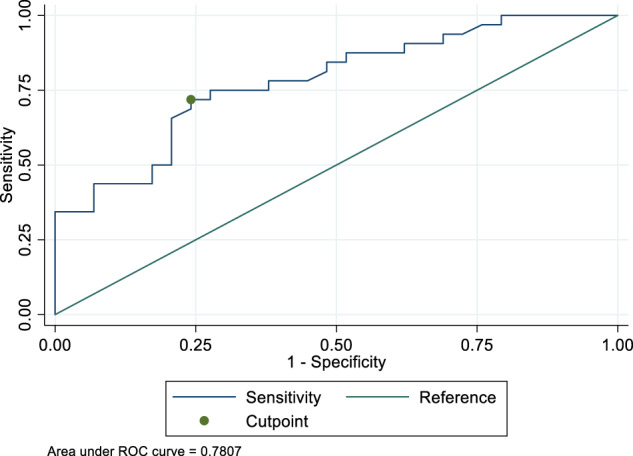
ROC curve: the threshold for the increased need for thyroxine was built by using 1.51 μg/kg/day as cut-off

In Group A, the median required T4 dose was 1.39 μg/kg/day (IQR = 1.26–1.47 μg/kg/day), being 23% lower than the dose of 1.71 μg/kg/day (IQR = 1.46–1.94 μg/kg/day) required by patients of group B (*p* < 0.0001) (Fig. 3). The distribution of patients requiring an increased dose was uneven inside the two pH-related groups. In fact, two patients out of 29 (7%) in the group A (normal pH) needed an increased T4 dose as compared with 23/32 patients (72%) in the group 2 (increased pH) (Fisher’s exact test *p* < 0.0001; PPV = 75%, NPV = 92%; RR = 9.38). The ROC curve (using 1.51 μg/kg/day as cut-off) revealed that the pH threshold that discriminated patients with increased thyroxine requirement was at 2.28, being the area under the curve by 78% (Fig. 4).

### The dose of thyroxine in relation with gastritis patterns

The need for thyroxine was then analyzed in relation with the histologic grading of mucosal gastric alterations. Overall, the histologic characterization of gastric mucosa revealed that: (a) 4/61 (6.6%) patients had minimal or no alterations (Group 0); (b) 8/61 (13.1%) patients had mild superficial chronic gastritis of the antrum (Group 1); (c) 20/61 (32.7%) patients had mild superficial chronic pangastritis (Group 2); (d) 7/61 (11.5%) had *H. Pylori*-related moderate/severe active superficial chronic pangastritis, (Group 3); (e) the remaining 22/61 patients (36.1%) showed gastric atrophy (Group 4). In this latter group, 18 out of 22 (82%) patients had an *H. Pylori*-related atrophic pangastritis while the remaining four patients (17%) had an autoimmune fundus/corpus-restricted atrophic gastritis. Table 2a describes the features of gastric function and immunology of the four groups. As expected from the literature [24–27, 36, 37], gastric pH was similar in the Groups 0, 1 and 2 (median pH 1.70 vs 1.39 vs 1.61, respectively; *p* = 0.1291). In contrast, Group 3 and 4 had significant higher gastric pH than 0 + 1 + 2 groups, again without differences between their median values (*p* = 0.1347). Therefore, statistical analysis was performed considering groups 0, 1 and 2 as a single entity (median pH 1.50; IQR = 1.30–1.96) and groups 3 and 4 as another single entity (median pH=6.54; IQR = 3.85–6.94), being the median pH of these single entities significantly different (*p* < 0.0001). In the groups 0 + 1 + 2, the median required dose of thyroxine was significantly lower (1.40 μg/kg/day; IQR = 1.27–1.49) than in the group 3 + 4 (1.69 μg/kg/day; IQR = 1.43–1.98) (*p* = 0.0025). In groups 0 + 1 + 2, only 6 out 32 (19%) of patients showed an increased need for thyroxine, as compared with 66% of those in groups 3 + 4 (*p* = 0.0003).

**Table 2:** **a** Gastric features of patients subdivided by histologic grading of gastric damage. **b** Characteristics of patients subdivided by positivity or negativity of *H. pylori* infection

(a)						
	*n*	pH	[H^+^] (mEq/l)	*H. pylori* infection	APCAb positivity	Gastrin (pg/ml)
Minimal or no alteration (GROUP 0)	4	1.70 (1-64-1.87)	64 (50–78)	0	0	22 (11–30)
Chronic superficial mild antral gastritis (GROUP 1)	8	1.39 (1.20–1.42)	83 (74–93)	0	1	40 (39–40)
Chronic superficial mild pangastritis (GROUP 2)	20	1.61 (1.30–2.09)	74 (54–83)	0	4	21(12–38)
Chronic active superficial moderate/severe pangastritis (GROUP 3)	7	2.44(2.18–6.87)	51 (1–63)	6	4	253 (144–320)
Gastric atrophy (GROUP 4)	22	6.57 (5.96–7.09)	5 (0–13)	17	18	460 (279–561)

(b)									
	Patients (*n*)	pH	[H^+^]	Age (years)	Weight (kg)	TSH (mU/l)	FT4 (ng/dl)	APCAb positivity (pos/neg)	Gastrin (pg/ml)
*H. pylori −*	37	1.62 (1.37–2.18)	73 (52–84)	51 (44–65)	66 (60–74)	1.23 (0.82–2.03)	1.25 (1.13–1.32)	8/29	39 (19–105)
*H. pylori* +	24	6.5 (3.80–6.98)	5 (1–28)	51 (40–67)	63 (59–76)	1.36 (1.05–1.80)	1.23 (1.15–1.34)	20/4	357 (243–525)
*p*		<0.0001	<0.0001	0.7009	0.6899	0.7686	0.8919	<0.0001	0.0005

### *H. pylori* infection and levothyroxine requirement

The possible role of *H.pylori* infection in the subsequent changes of gastric pH, prompted us to analyze whether its distribution may affect the daily dose of oral T4. In the whole sample the prevalence of *H.pylori* infection was 39.5% (24/61 patients). Interestingly, no infected patients were recognized in the first three groups (0–2; *n* = 32) while 24 out of 29 patients (83%) from groups 3 and 4 had histological evidence of *H.pylori* infection. Amongst the 37 *H.pylori*–negative, only nine patients showed a pH >2 while, in the group *H.pylori*–positive, only 2/24 had pH ≤ 2. It follows that the median pH of gastric juice was significantly higher in *H.pylori*–positive than in *H.pylori*–negative patients (*p* < 0.0001) (Table 2b) as well as the median daily requirement of thyroxine that was 1.71 μg/kg/day (IQR = 1.44–1.99) in *H. pylori-*positive vs. 1.42 μg/kg/day (IQR = 1.27–1.51) in *H. pylori-*negative patients (*p* = 0.0027).

### Multivariate analysis

Beside the expected and recognized role of BMI, the results of multivariate analysis revealed that gastric pH was the far more important independent variable in determining the effective dose of T4 (*p* = 0.001) (Table 3).

**Table 3:** Results of the multiple linear regression to identify predictors of thyroxine dose

Variable	Coefficient	SE^a^	pV
pH (continue)	0.067	0.020	0.001
Age (years, continue)	−0.003	0.002	0.187
Sex (0 = male; 1=female)	−0.154	0.085	0.071
BMI (kg/m^2^)	0.030	0.005	0.001
Gastritis pattern			
No alteration	Ref.	–	–
Antral superficial gastritis	−0.108	0.141	0.433
Superficial chronic pangastritis	0.007	0.109	0.950
Active superficial pangastritis	−0.091	0.169	0.592
Atrophic chronic pangastritis	−0.195	0.166	0.240
TSH (mU/l, continue)	0.016	0.035	0.651
*Helicobacter pylori* (0 = absent; 1 = present)	0.045	0.094	0.632

^a^Standard error

## Discussion

Our data represent the first direct evidence of the role of gastric pH in reaching the pharmacologic thyroid homeostasis. In fact, this in vivo study revealed that the effective dose of thyroxine linearly correlates with gastric juice pH. Hydrogen ions concentration in gastric juice is, in fact, the far more important independent variable, beside the BMI, in determining the minimal effective dose of T4. Increased need for thyroxine grows up with the increased gastric pH and reaches the higher doses of T4 in patients with the highest degree of oxyntic glands atrophy and achlorhydria.

The overall mechanism that impairs intestinal absorption of thyroxine in hypo–achlorhydric patients is matter of debate. Up to now, some evidence indirectly suggested the involvement of pH in shaping the minimal effective oral dose of T4 in vivo [20, 21, 28]. A growing body of in vitro evidence indicates that, similarly to other drugs, the thyroxine molecule is very sensitive to environmental pH variations [9, 10, 29]. In murine models, changes in blood pH may affect the partition between the fast exchanging pool of thyroxine between tissues and the intravascular milieu [40] and several membrane transporters of thyroxine appeared to be sensitive to pH changes [41]. Moreover, Pabla and Coll. have shown that the dissolution of tablet thyroxine exceeded 85% only at “normal pH” (<2.0) and rapidly decreases at medium pH > 3 [42]. To note, the T4 sodium salt in tablet formulation, before being absorbed, undergoes disaggregation and dissolution in the stomach [17]. Whether thyroxine, at intestinal level, would be absorbed as naïve form or as a sodium salt is still unknown. In the hypothesis that the naïve form would be the preferred one at the absorption site, thus our data would indicate that, in a hypo–/achlorhydric gastric environment, sodium levothyroxine salt may remain partly undissociated and this event may impair the efficiency of subsequent intestinal absorption [20]. Furthermore, a structural study on thyroxine polymorphs demonstrates that the variations of pH in the medium may modify the crystalline conformation of T4 molecule and its physical properties, thereby affecting its solubility [10].

The altered gastric pH may affect the small intestine juice pH [43, 44], possibly affecting the activity of the putative T4 intestinal transporters, being some of them pH-dependent structures [41]. Besides, reduced gastric acid secretion may affect luminal ion concentration and pH and could damage the gut microbiota of upper gastrointestinal tract and favors bacterial overgrowth [45, 46]. On this ground, it is worth to note that a variation of microbial composition at the upper intestinal absorption site may interfere with deconjugation of bile acid [47] leading to changes in the thyroxine enterohepatic recycling [48, 49].

How specific is the effect of gastric pH changes in determining the minimal effective dose of thyroxine? The answer comes from drugs certainly interacting with gastric acidity [29]. From a pharmacologic standpoint, the role of gastric pH was highlighted by Seng et al., who suggested that a bioequivalence in healthy volunteers might differ from the one in patients bearing altered gastric pH [50]. In a clinical study, a more efficient absorption of thyroxine has been observed when vitamin C was simultaneously taken in patients bearing both hypothyroidism and gastritis [51]. In these patients with impaired acid secretion, ascorbic acid may lower gastric pH thus facilitating the T4 absorption process [51]. More recently, a pharmacokinetic study in healthy euthyroid subjects by Chon et al. [52] showed that simultaneous milk ingestion decreases oral levothyroxine absorption. Interestingly, the pH of cow’s milk is ~6.6 and it contains about 1 g of calcium per liter. Both these factors affect the pH–sensitive fraction of T4 absorption [53]. This implies that any food altering the gastric pH during the first hour from the ingestion of levothyroxine [12, 16, 19, 20] may prevent the optimal absorption of LT4, leading to an increased need for therapeutic dose of the hormone [54].

Besides these nutritional interferences, it should be noted that the presence of *H.pylori* infection may also act as a confounding factor since:

- gastric juice pH may vary according to the phase of infection. In fact, chronic infection with *H.pylori* may feature either decreased or increased acid secretion, along with the distribution and/ or the severity of gastritis [55].
- while autoimmune atrophic gastritis is substantially irreversible, *H.pylori*-related atrophic gastritis is reversible in about 20% of cases [56].

It follows that, when an *H. pylori* infection occurs in patients treated with levothyroxine, an accurate follow-up of thyroid homeostasis is recommended until eradication has been confirmed.

From a therapeutic standpoint, recent evidence suggested that novel pharmacologic preparations of thyroxine might be more effective than levothyroxine tablet formulation, in patients with impaired gastric acidic output [57]. Indeed, pilot studies have provided evidence that switching from tablet formulation to liquid [58] or softgel T4 preparations [59], was associated to superior pharmacokinetic parameters in patients with impaired gastric acidity [50] and to a lower therapeutic dose in patients bearing gastric disorders such as *H.pylori* infection [60]. These novel preparations, as compared to tablet, require no disaggregation process and showed a better dissolution profile (softgel T4) [42] or even no need for dissolution (liquid T4) [57].

The clinical relevance of the present findings stems from the following:

- the high frequency of gastric disorders in general population and in thyroid autoimmune disorders [61–63],
- the prescription rate of proton pump inhibitors [2],
- the impact of food co-ingestion with oral thyroxine in term of absorption efficiency [12, 52].

Last, but not least, the use of healthcare resources is increased in patients experiencing multiple levothyroxine dose adjustments and an increased costs for the health systems has been proven [64].

A major limitation of this study comes from the number of patients which however is hardly possible to overcome. In fact, the invasive procedure required to obtain gastric juice pH and the limited number of patients with stably increased pH prevent from designing studies in large populations.

In conclusion, our data highlight a key role of gastric pH in vivo in shaping the minimal effective dose of thyroxine in hypothyroid patients. Therefore, when prescribing oral thyroxine, physicians should keep in mind all the physiologic, nutritional, pharmacologic, and pathologic conditions that may impair the gastric acid secretion.

## References

[1] Ladenson PW (2016). Precision medicine comes to thyroidology. J. Clin. Endocrinol. Metab..

[2] http://clincalc.com/DrugStats/Top200Drugs.aspx. The Top 200 of 2021 provided by the ClinCalc DrugStats Database. Accessed 15 Mar 2021

[3] Jonklaas J, Bianco AC, Bauer AJ, Burman KD, Cappola AR, Celi FS, Cooper DS, Kim BW, Peeters RP, Rosenthal MS, Sawka AM (2014). American Thyroid Association Task Force on Thyroid Hormone Replacement. Guidelines for the treatment of hypothyroidism: prepared by the American Thyroid Association task force on thyroid hormone replacement. Thyroid.

[4] Garber JR, Cobin RH, Gharib H, Hennessey JV, Klein I, Mechanick JI, Pessah-Pollack R, Singer PA, Woeber KA (2012). American Association Of Clinical Endocrinologists And American Thyroid Association Taskforce On Hypothyroidism In Adults. Clinical practice guidelines for hypothyroidism in adults: cosponsored by the American Association of Clinical Endocrinologists and the American Thyroid Association. Endocr. Pract..

[5] Biondi B, Wartofsky L (2014). Treatment with thyroid hormone. Endocr. Rev..

[6] Eligar V, Taylor PN, Okosieme OE, Leese GP, Dayan CM (2016). Thyroxine replacement: a clinical endocrinologist’s viewpoint. Ann. Clin. Biochem..

[7] Biondi B, Cooper DS (2018). Subclinical Hyperthyroidism. N. Engl. J. Med..

[8] Biondi B (2012). Natural history, diagnosis and management of subclinical thyroid dysfunction. Best. Pract. Res. Clin. Endocrinol. Metab..

[9] Chemburkar SR, Deming KC, Reddy RE (2010). Chemistry of thyroxine: an historical perspective and recent progress on its synthesis. Tetrahedron.

[10] Mondal S, Mugesh G (2015). Structure elucidation and characterization of different thyroxine polymorphs. Angew. Chem. Int. Ed. Engl..

[11] Hays MT (1991). Localization of human thyroxine absorption. Thyroid.

[12] Wenzel KW, Kirschsieper HE (1977). Aspects of the absorption of oral L-thyroxine in normal man. Metabolism.

[13] Younis IR, Ahmed MA, Burman KD, Soldin OP, Jonklaas J (2018). Stable isotope pharmacokinetic studies provide insight into effects of age, sex, and weight on levothyroxine metabolism. Thyroid.

[14] Santini F, Pinchera A, Marsili A, Ceccarini G, Castagna MG, Valeriano R, Giannetti M, Taddei D, Centoni R, Scartabelli G, Rago T, Mammoli C, Elisei R, Vitti P (2005). Lean body mass is a major determinant of levothyroxine dosage in the treatment of thyroid diseases. J. Clin. Endocrinol. Metab..

[15] Bolk N, Visser TJ, Nijman J, Jongste IJ, Tijssen JGP, Berghout A (2010). Effects of evening vs morning levothyroxine intake: a randomized double-blind crossover trial. Arch. Intern. Med..

[16] Pang X, Pu T, Xu L, Sun R (2020). Effect of l-LT4 administration before breakfast vs at bedtime on hypothyroidism: a meta-analysis. Clin. Endocrinol..

[17] Virili C, Antonelli A, Santaguida MG, Benvenga S, Centanni M (2019). Gastrointestinal malabsorption of thyroxine. Endocr. Rev..

[18] Centanni M, Benvenga S, Sachmechi I (2017). Diagnosis and management of treatment-refractory hypothyroidism: an expert consensus report. J. Endocrinol. Investig..

[19] Benvenga S, Bartolone L, Squadrito S, Lo Giudice F, Trimarchi F (1995). Delayed intestinal absorption of levothyroxine. Thyroid.

[20] Centanni M, Gargano L, Canettieri G, Viceconti N, Franchi A, Delle Fave G, Annibale B (2006). Thyroxine in goiter, Helicobacter pylori infection, and chronic gastritis. N. Engl. J. Med..

[21] Lahner E, Virili C, Santaguida MG, Annibale B, Centanni M (2014). Helicobacter pylori infection and drugs malabsorption. World J. Gastroenterol..

[22] Khraisha OS, Al-Madani MM, Peiris AN, Paul TK (2015). Gastroparesis a novel cause of persistent thyroid stimulating hormone elevation in hypothyroidism. J. LA State Med. Soc..

[23] Sachmechi I, Reich DM, Aninyei M, Wibowo F, Gupta G, Kim PJ (2007). Effect of proton pump inhibitors on serum thyroid-stimulating hormone level in euthyroid patients treated with levothyroxine for hypothyroidism. Endocr. Pract..

[24] Lu PJ, Hsu PI, Chen CH, Hsiao M, Chang WC, Tseng HH, Lin KH, Chuah SK, Chen HC (2010). Gastric juice acidity in upper gastrointestinal diseases. World J. Gastroenterol..

[25] Rugge M, Genta RM (2005). Staging and grading of chronic gastritis. Hum. Pathol..

[26] Sung J, Kim N, Lee J, Hwang YJ, Kim HW, Chung JW, Kim JW, Lee DH (2018). Associations among gastric juice pH, atrophic gastritis, intestinal metaplasia and *Helicobacter pylori* infection. Gut Liver.

[27] Sipponen PI, Maaroos HI (2015). Chronic gastritis. Scand. J. Gastroenterol..

[28] Cellini M, Santaguida MG, Virili C, Capriello S, Brusca N, Gargano L, Centanni M (2017). Hashimoto’s thyroiditis and autoimmune gastritis. Front Endocrinol..

[29] Mitra A, Kesisoglou F (2013). Impaired drug absorption due to high stomach pH: a review of strategies for mitigation of such effect to enable pharmaceutical product development. Mol. Pharm..

[30] Markl D, Zeitler JA (2017). A review of disintegration mechanisms and measurement techniques. Pharm. Res..

[31] Won CM (1992). Kinetics of degradation of levothyroxine in aqueous solution and in solid state. Pharm. Res..

[32] Talley NJ, Ford AC (2015). Functional dyspepsia. N. Engl. J. Med..

[33] Sibilla R, Santaguida MG, Virili C, Gargano L, Nardo S, Della Guardia M, Viceconti N, Franchi A, Centanni M (2008). Chronic unexplained anaemia in isolated autoimmune thyroid disease or associated with autoimmune related disorders. Clin. Endocrinol..

[34] Virili C, Bassotti G, Santaguida MG, Iuorio R, Del Duca SC, Mercuri V, Picarelli A, Gargiulo P, Gargano L, Centanni M (2012). Atypical celiac disease as cause of increased need for thyroxine: a systematic study. J. Clin. Endocrinol. Metab..

[35] Cellini M, Santaguida MG, Gatto I, Virili C, Del Duca SC, Brusca N, Capriello S, Gargano L, Centanni M (2014). Systematic appraisal of lactose intolerance as cause of increased need for oral thyroxine. J. Clin. Endocrinol. Metab..

[36] Dixon MF, Genta RM, Yardley JH, Correa P (1996). Classification and grading of gastritis. The updated Sydney System. International Workshop on the Histopathology of Gastritis, Houston 1994. Am. J. Surg. Pathol..

[37] Hunt RH, Camilleri M, Crowe SE, El-Omar EM, Fox JG, Kuipers EJ, Malfertheiner P, McColl KE, Pritchard DM, Rugge M, Sonnenberg A, Sugano K, Tack J (2015). The stomach in health and disease. Gut.

[38] Di Mario F, Goni E (2014). Gastric acid secretion: changes during a century. Best Pract. Res. Clin. Gastroenterol..

[39] Annibale B, Capurso G, Lahner E, Passi S, Ricci R, Maggio F, Delle Fave G (2003). Concomitant alterations in intragastric pH and ascorbic acid concentration in patients with Helicobacter pylori gastritis and associated iron deficiency anaemia. Gut.

[40] Gordon A, Coutsoftides T (1971). The effect of blood pH on the acute distribution of thyroxine in the rat. Endocrinology.

[41] Hennemann G, Docter R, Friesema EC, de Jong M, Krenning EP, Visser TJ (2001). Plasma membrane transport of thyroid hormones and its role in thyroid hormone metabolism and bioavailability. Endocr. Rev..

[42] Pabla D, Akhlaghi F, Zia H (2009). A comparative pH-dissolution profile study of selected commercial levothyroxine products using inductively coupled plasma mass spectrometry. Eur. J. Pharm. Biopharm..

[43] Litou C, Vertzoni M, Goumas C, Vasdekis V, Xu W, Kesisoglou F, Reppas C (2016). Characteristics of the human upper gastrointestinal contents in the fasted state under hypo- and a-chlorhydric gastric conditions under conditions of typical drug - drug interaction studies. Pharm. Res..

[44] Cao L, Yuan Z, Liu M, Stock C (2020). (Patho-)Physiology of Na^+^/H^+^ exchangers (NHEs) in the Digestive system. Front. Physiol..

[45] Dash NR, Khoder G, Nada AM, Al Bataineh MT (2019). Exploring the impact of Helicobacter pylori on gut microbiome composition. PLoS ONE.

[46] Bruno G, Zaccari P, Rocco G, Scalese G, Panetta C, Porowska B, Pontone S, Severi C (2019). Proton pump inhibitors and dysbiosis: Current knowledge and aspects to be clarified. World J. Gastroenterol..

[47] Shindo K, Machida M, Fukumura M, Koide K, Yamazaki R (1998). Omeprazole induces altered bile acid metabolism. Gut.

[48] Hazenberg MP, de Herder WW, Visser TJ (1988). Hydrolysis of iodothyronine conjugates by intestinal bacteria. FEMS Microbiol. Rev..

[49] Virili C, Centanni M (2017). “With a little help from my friends” - The role of microbiota in thyroid hormone metabolism and enterohepatic recycling. Mol. Cell. Endocrinol..

[50] Seng Yue C, Benvenga S, Scarsi C, Loprete L, Ducharme MP (2015). When bioequivalence in healthy volunteers may not translate to bioequivalence in patients: differential effects of increased gastric ph on the pharmacokinetics of levothyroxine capsules and tablets. J. Pharm. Pharm. Sci..

[51] Jubiz W, Ramirez M (2014). Effect of vitamin C on the absorption of levothyroxine in patients with hypothyroidism and gastritis. J. Clin. Endocrinol. Metab..

[52] Chon DA, Reisman T, Weinreb JE, Hershman JM, Leung AM (2018). Concurrent milk ingestion decreases absorption of levothyroxine. Thyroid.

[53] Zamfirescu I, Carlson HE (2011). Absorption of levothyroxine when coadministered with various calcium formulations. Thyroid.

[54] Virili C, Brusca N, Capriello S, Centanni M (2021). Levothyroxine therapy in gastric malabsorptive disorders. Front. Endocrinol..

[55] Schubert ML, Peura DA (2008). Control of gastric acid secretion in health and disease. Gastroenterology.

[56] Vannella L, Lahner E, Bordi C, Pilozzi E, Di Giulio E, Corleto VD, Osborn J, Delle Fave G, Annibale B (2011). Reversal of atrophic body gastritis after *H. pylori* eradication at long-term follow-up. Dig. Liver Dis..

[57] Virili C, Trimboli P, Centanni M (2019). Novel thyroxine formulations: a further step toward precision medicine. Endocrine.

[58] Fallahi P, Ferrari SM, Ruffilli I, Antonelli A (2016). Reversible normalisation of serum TSH levels in patients with autoimmune atrophic gastritis who received L-T4 in tablet form after switching to an oral liquid formulation: a case series. BMC Gastroenterol..

[59] Santaguida MG, Virili C, Del Duca SC, Cellini M, Gatto I, Brusca N, De Vito C, Gargano L, Centanni M (2015). Thyroxine softgel capsule in patients with gastric-related T4 malabsorption. Endocrine.

[60] Ribichini D, Fiorini G, Repaci A, Castelli V, Gatta L, Vaira D, Pasquali R (2017). Tablet and oral liquid L-thyroxine formulation in the treatment of naïve hypothyroid patients with Helicobacter pylori infection. Endocrine.

[61] Robertson HM, Narayanaswamy AK, Pereira O, Copland SA, Herriot R, McKinlay AW, Bevan JS, Abraham P (2014). Factors contributing to high levothyroxine doses in primary hypothyroidism: an interventional audit of a large community database. Thyroid.

[62] Castellana M, Castellana C, Giovanella L, Trimboli P (2020). Prevalence of gastrointestinal disorders having an impact on tablet levothyroxine absorption: should this formulation still be considered as the first-line therapy?. Endocrine.

[63] Lahner E, Intraligi M, Buscema M, Centanni M, Vannella L, Grossi E, Annibale B (2008). Artificial neural networks in the recognition of the presence of thyroid disease in patients with atrophic body gastritis. World J. Gastroenterol..

[64] Ernst FR, Barr P, Elmor R, Sandulli W, Thevathasan L, Sterman AB, Goldenberg J, Vora K (2017). The economic impact of levothyroxine dose adjustments: the CONTROL HE study. Clin. Drug. Investig..

